# Synthesis and biological evaluation of some novel 1-substituted fentanyl analogs in Swiss albino mice

**DOI:** 10.2478/intox-2014-0013

**Published:** 2014-11-15

**Authors:** Shiv Kumar Yadav, Chandra Kant Maurya, Pradeep Kumar Gupta, Ajai Kumar Jain, Kumaran Ganesan, Rahul Bhattacharya

**Affiliations:** 1 Pharmacology and Toxicology Division, Defence Research and Development Establishment, Jhansi Road, Gwalior 474 002, (M.P.), India; 2 Synthetic Chemistry Division, Defence Research and Development Establishment, Jhansi Road, Gwalior 474 002, (M.P.), India; 3 School of Studies in Zoology, Jiwaji University, Gwalior 474 011, (M.P.), India

**Keywords:** fentanyl analogs, opioids, synthesis, toxicity, efficacy

## Abstract

Fentanyl [*N*-(1-phenethyl-4-piperidinyl)propionanilide] is a potent opioid analgesic agent, but a has narrow therapeutic index. We reported earlier on the synthesis and bioefficacy of fentanyl and its 1-substituted analogs (**1–4**) in mice. Here we report the synthesis and biological evaluation of four additional analogs, viz. *N*-isopropyl-3-(4-(*N*-phenylpropionamido)piperidin-1-yl)propanamide (**5**), *N*-*t*-butyl-3-(4-(*N*-phenylpropionamido)piperidin-1-yl)propanamide (**6**), isopropyl 2-[4-(*N*-phenylpropionamido)piperidin-1-yl]propionate (**7**) and *t*-butyl 2-[4-(*N*-phenylpropionamido)piperidin-1-yl]propionate (**8**). The median lethal dose (LD_50_) determined by intravenous, intraperitoneal and oral routes suggests these analogs to be comparatively less toxic than fentanyl. On the basis of observational assessment on spontaneous activities of the central, peripheral, and autonomic nervous systems, all the analogs were found to be similar to fentanyl. Naloxone hydrochloride abolished the neurotoxic effects of these analogs, thereby ascertaining their opioid receptor-mediated effects. All the analogs displayed significant analgesic effects, measured by formalin-induced hind paw licking and tail immersion tests at their respective median effective dose (ED_50_). They also exhibited 8–12 fold increase in therapeutic index over fentanyl. However, **5** and **6** alone produced lower ED_50_ (20.5 and 21.0 µg/kg, respectively) and higher potency ratio (1.37 and 1.33, respectively) compared to fentanyl. They could thus be considered for further studies on pain management.

## Introduction

Severe and chronic pain conditions are usually alleviated by narcotic (opioid) analgesics like morphine (Vogel, 2002). However, their beneficial effects are often obscured by unwanted effects like constipation, nausea, vomiting, etc. (Kalso *et al*., 2004). Fentanyl [*N*-(1-phenethyl-4-piperidinyl)propionanilide] is a fully synthetic opioid analgesic that has found several clinical applications because of its rapid onset of action and good safety margin. As an analgesic, it is several times more potent than morphine (Mather, 1983; Mićović *et al*., 2000; Van Nimmen *et al*., 2004). Fentanyl is characterized by high lipophilicity and therefore it penetrates easily the central nervous system (CNS) to interact with *µ*-opioid receptors (mu opioid receptors; MOR), resulting in inhibition of pain neurotransmission (Kieffer, 1995; Mayes & Ferrone, 2006; Kieffer & Evans, 2009). This pharmacological characteristic of fentanyl prompted several workers to synthesize numerous new analogs of fentanyl including sufentanil, alfentanil, remifentanil, lofentanil, etc. (Lemmens, 1995; Scholz *et al*., 1996).

Although, fentanyl and many of its analogs have exhibited marked antinociceptive activity, they are not free from certain undesirable effects like muscular rigidity, respiratory depression, tolerance and addiction (Vučović *et al*., 2000). Toxic signs elicited by fentanyl are somewhat similar to those produced by morphine, like increased spontaneous motor activity, circling, straub tail reaction, mydriasis, hypertonia, tactile hypersensitivity, convulsions, and respiratory depression leading to death (Gardocki & Yelnosky, 1964). In order to discover an analgesic with improved pharmacodynamics and pharmacokinetics, extensive efforts have been made in synthesizing several new fentanyl analogs and determining their structure activity relationship (SAR) (Casy and Parfitt, 1986; Bagley *et al*., 1991; Portoghese, 1992; Bi-Yi *et al*., 1999; Gerak *et al*., 1999). Such studies can lead to an ideal analgesic with greater potency, duration of action and safety (Higashikawa & Suzuki, 2008).

We also reported on the synthesis and bioefficacy of fentanyl and its 1-substituted analogs (**1**–**4**), where the phenethyl chain of fentanyl was replaced by alkyl, ethereal and nitrile functional groups (Gupta *et al*., 2013). Compared to fentanyl and its four analogs, **2** exhibited the lowest median effective dose (ED_50_) and highest potency ratio. Therefore, with an objective to synthesize more compounds with still lower ED_50_ and higher potency ratio, we report here the synthesis and biological evaluation of four more 1-substituted analogs of fentanyl, viz., *N*-isopropyl-3-(4-(*N*-phenylpropionamido)piperidin-1-yl)propanamide (**5**), N-t-butyl-3-(4-(*N*-phenylpropionamido)piperidin-1-yl)propanamide (**6**), isopropyl 2-[4-(*N*-phenylpropionamido)piperidin-1-yl]propionate (**7**) and t-butyl 2-[4-(*N*-phenylpropionamido)piperidin-1-yl]propionate (**8**). In the present study, the phenethyl chain of fentanyl was replaced by different functional groups, viz. *N*-isopropyl propanamide, *N*-t-butyl propanamide, isopropyl propionate, and t-butyl propionate moieties. The median lethal dose (LD_50_), opioid receptor-mediated activity, antinociceptive effects, ED_50_, and analgesic potency ratio of **5–8** were determined and compared with fentanyl as reported earlier in our publication (Gupta *et al*., 2013).

## Materials and methods

### Chemistry

All the chemicals used in the present study were of the highest purity. Acrylonitrile (CAS 107-13-1), 2-bromopropionyl chloride (CAS 7148-74-5), dimethyl sulfoxide (DMSO; CAS 67-68-5) and naloxone hydrochloride (CAS 51481-60-8) were purchased from Sigma-Aldrich Inc. (St. Louis, USA). Formaldehyde (CAS: 50-00-0) was procured from Merck (Mumbai, India), isopropanol (CAS 67-63-0) was obtained from Rankem (New Delhi, India), and tert-butanol (CAS 75-65-0) was from Acros (NJ, USA).

#### Synthesis of fentanyl analogs

All the 1-substituted fentanyl analogs were synthesized from a common precursor (*N*-(4-piperidinyl)propionanilide), prepared by the procedure reported earlier (Gupta *et al*., 2013), and they were found to be >98% pure. Structure and yield of fentanyl analogs are shown in Table 1.

**Scheme 1:**
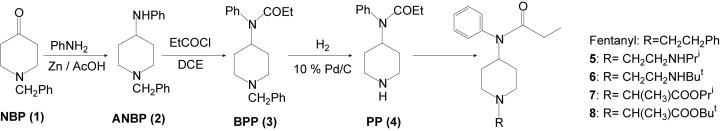
Synthetic route for fentanyl and its analogs.

**Table 1:** Structure and yield of fentanyl analogs.

	Compound name	Substituent (R)	Molecular weight	Yield (%)	Structure
**5**	*N*-isopropyl-3-(4-(*N*-phenylpropionamido)piperidin-1-yl)propanamide	-CH_2_CH_2_NHPr^i^	345	60	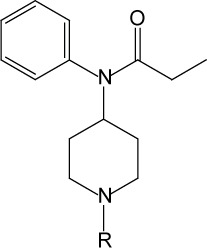
**6**	*N*-t-butyl-3-(4-(*N*-phenylpropionamido)piperidin-1-yl)propanamide	-CH_2_CH_2_NHBu^t^	359	65		
**7**	Isopropyl 2-[4-(*N*-phenylpropionamido)piperidin-1-yl]propionate	-CH(CH_3_)COOPr^i^	346	76		
**8**	*t*-butyl 2-[4-(*N*-phenylpropionamido)piperidin-1-yl]propionate	-CH(CH_3_)COOBu^t^	360	74		

#### 4-Anilino-N-benzylpiperidine (ANBP; **2**)

**Figure:**
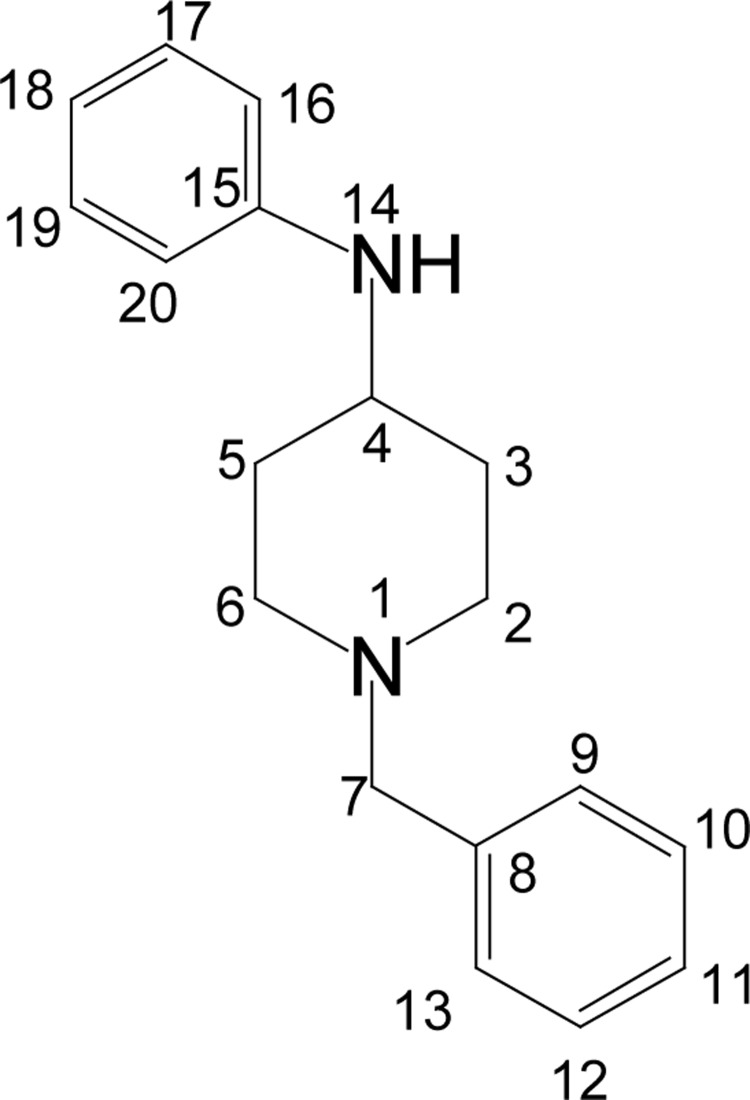


To a mixture of *N*-benzyl-4-piperidone (0.10 moles), aniline (0.10 moles) and activated zinc (0.40 moles), 90% aqueous acetic acid (1.60 moles) was added portion wise, and the resulting mixture was allowed to stir at room temperature for 24h and at 60–70°C in water bath for another 12h. After completion of the reaction, the content of the flask was diluted with methanol and filtered. The filtrate was concentrated under vacuum and then neutralized with 30% ammonium hydroxide solution till pH10. The crude product was collected by filtration and recrystallized with petroleum ether (60–80°C) as colorless solid. Yield 85%, mp 82–83°C. IR (KBr) v_max_ 3440, 3250, 3025, 2930, 2848, 1605, 1526, 1492, 1371, 1317, 1250, 1085, 975, 862, 750, 690 cm^−1^; ^1^H NMR [CDCl_3_, 400 MHz): δ=1.50 (dq, 2H, H-3_ax_, 5_ax_), 2.10 (bd, 2H, H-3_eq_, 5_eq_), 2.30 (bt, 2H, H-2_ax_, 6_ax_), 2.60 (s, 2H, H-7), 2.90 (bd, 2H, H-2_eq_, 6_eq_), 3.35 (m, 1H, H-4), 3.50 (sbr, 1H, PhNH), 7.10–7.40 (m, 10×Ar-H); ^13^C NMR [CDCl_3_, 100 MHz): d=32.7 (CH_2_, C-3, 5), 50.3 (CH_2_, C-2, 6), 52.2 (CH, C-4), 62.7 (CH_2,_ C-7), 146.0 (CH, C-15), 138.6 (CH, C-8), 129.5 (CH, C-9, 13, 17, 19), 128.0 (CH, C-10, 12), 127.6 (CH, 11), 116.5 (CH, C-18), 113.0 (CH, C-16, 20), 174.8 (CO); EI-MS m/z (pos): 267 [M+1], 266 [M], 173, 158, 146, 132, 118, 91, 82, 65; Anal. Calcd. for C_21_H_26_N_2_O: C, 78.22; H, 8.13; N, 8.69. Found: C, 78.15; H, 8.00; N, 8.60.

#### N-(1-benzyl-4-piperidinyl)propionanilide (BPP; **3**)

**Figure:**
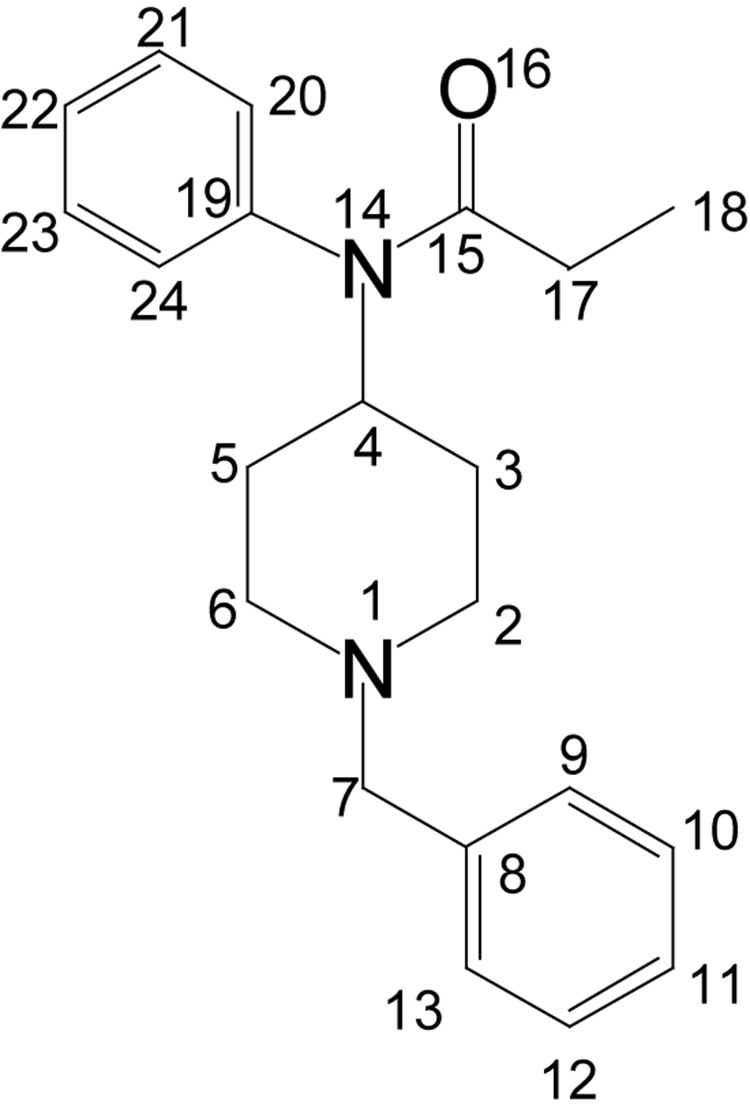


To the solution of ANBP (0.08 moles), 150 ml of 1,2-dichloroethane, propionyl chloride (0.24 moles) was added drop-wise and the resulting mixture was stirred at ambient temperature for 2 h. After completion of the reaction, the reaction mixture was poured slowly into 4% aqueous sodium hydroxide solution with continuous stirring. The resulting alkaline solution was extracted with dichloromethane, the organic phase was dried over anhydrous sodium sulphate and concentrated under reduced pressure to get the crude product. It was purified as its hydrochloride salt. Colorless crystals, yield 25.78g (0.072 moles, 90%), mp 232–233°C (ethyl acetate). The corresponding free base was obtained by decomposition of its hydrochloride salt with 20% sodium hydroxide solution followed by recrystallization from petroleum ether (60–80°C), colorless compound, yield 23.18 g (0.072 moles, 90%), mp 72–73°C. IR (KBr) ν_max_ 3430, 2941, 2822, 1659 (C=O), 1495, 1370, 1260 (C-N Str), 1150, 1090, 705 cm^−1^; ^1^H NMR [CDCl_3_, 400 MHz): d=0.94 (t, 3H, H-18), 1.30–1.40 (m, 2H, H-3_ax_, 5_ax_), 1.70–1.80 (m, 2H, H-3_eq_, 5_eq_), 1.85 (q, 2H, H-17), 2.10 (m, 2H, H-2_ax_, 6_ax_), 2.65 (m, 2H, H-2_eq_, 6_eq_), 3.30 (t, 2H, H-7), 4.58–4.67 (m, 1H, H-4), 7.10–7.30 (m, 10 × Ar-H); ^13^C NMR [CDCl_3_, 100 MHz): d=9.8 (CH_3_, C-18), 27.8 (CH_2_, C-17), 30.1 (CH2, C-3, 5), 52.4 (CH2, C-2, 6), 53.0 (CH, C-4), 62.5 (CH2, C-7), 126.4 (CH, C-20, 24), 128.1 (CH, C-22), 129.0 (CH, C-11), 130.4 (CH, C-10, 12), 130.7 (CH, C-21, 23), 135.0 (CH, C-9, 13), 138.3 (CH, C-8), 140.8 (CH, C-19); EI-MS m/z (pos): 323 [M+1]^+^, 322 [M]^+^, 265, 173, 158, 146, 132, 118, 91, 82, 77, 65, 57; Anal. Calcd. for C_21_H_26_N_2_O: C, 78.22; H, 8.13; N, 8.69. Found: C, 78.15; H, 8.00; N, 8.60.

#### N-(4-piperidinyl)propionanilide (PP; **4**)

**Figure:**
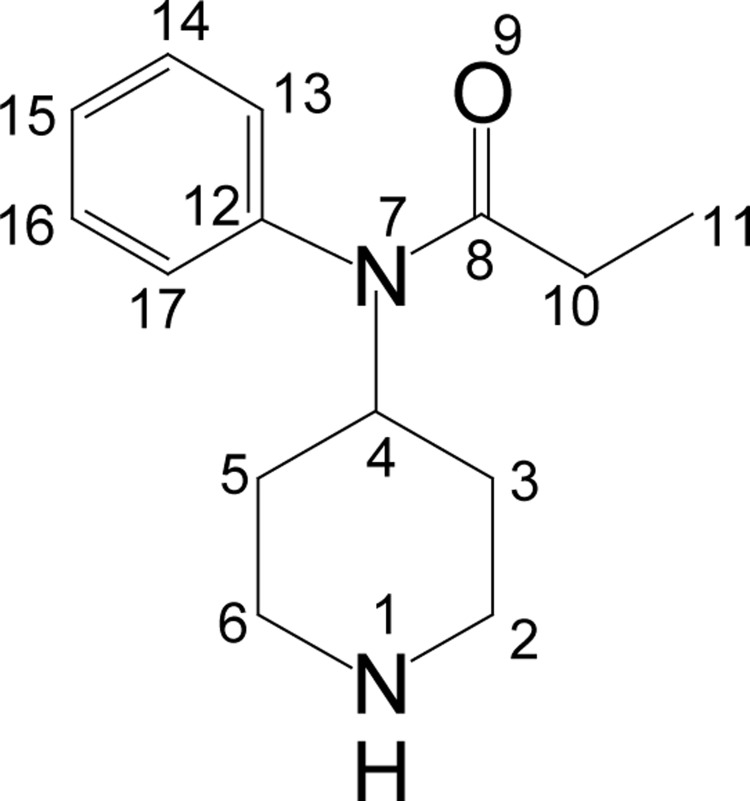


A solution of BPP (0.07 moles) in 125 ml methanol-acetic acid mixture (3:2) was taken in a 250 ml thick-walled hydrogenation vessel containing 10% palladium on charcoal catalyst (10% w/w). The hydrogen gas was then purged into the vessel using Parr apparatus at 50°C. When no further amount of hydrogen was consumed, the vessel was removed and the contents were filtered through celite. The filtrate was concentrated on rotary evaporator and the residue was treated with 20% aqueous sodium hydroxide solution. The aqueous solution was extracted with ethyl acetate, dried over anhydrous sodium sulphate and the solvent was removed under vacuum. The crude compound thus obtained was recrystallized with petroleum-ether (40–60°C), yield 14.62g (0.063 moles, 90%), mp 81–83°C. IR (KBr) ν_max_ 3370, 3029, 2942, 2827, 1656, 1590, 695 cm^−1^; ^1^H NMR [CDCl_3_, 400 MHz): δ=0.94 (t, 3H, H-11), 1.25 (dq, 2H, H-3_ax_, 5_ax_), 1.55 (sbr, 1H, NH), 1.75 (bd, 2H, H-3_eq_, 5_eq_), 1.90 (q, 2H, H-10), 2.10 (bt, 2H, H-2_ax_, 6_ax_), 3.00 (bd, 2H, H-2_eq_, 6_eq_), 4.65–4.75 (m, 1H, H-4), 7.03 (d, 2×Ar-H), 7.05 (d, 1×Ar H), 7.35 (m, 2×Ar-H); ^13^C NMR [CDCl_3_, 100 MHz): δ=9.5 (CH_3_, C-11), 28.4 (CH_2_, C-10), 31.8 (CH_2_, C-3, 4), 46.0 (CH_2_, C-2, 3), 52.2 (CH, C-4), 128.1 (CH, C-13, 17), 129.1 (CH, C-15), 130.3 (CH, C-14, 16), 138.8 (CH, C-12), 173.2 (CO); EI-MS m/z (pos): 175, 146, 132, 118, 82, 77, 68, 57, 55; Anal. Calcd. for C_14_H_20_N_2_O: C, 72.38; H, 8.68; N, 12.06. Found: C, 72.05; H, 8.50; N, 11.99.

#### Synthesis of **5** and **6**

In a three neck round bottom flask, sulfuric acid (150 mmol) was heated to 45°C and a mixture of acrylonitrile (75 mmol) and isopropanol/tert-butanol (74 mmol) was added drop-wise. Thereafter, the reaction mixture was stirred at 60°C for 3 h to complete the reaction. The reaction mixture was then poured cautiously into ice cooled water with continuous stirring. The white precipitate was filtered off, washed well with plenty of water and dried under vacuum to obtain *N*-isopropyl/ tert-butyl acrylamide. To a solution of *N*-isopropyl or t-butyl acrylamide (10 mmol) and *N*-(4-piperidinyl)propionanilide (10 mmol) in acetonitrile, silica gel (1.0 g) was added and the heterogeneous mixture was stirred at 80°C till completion of the reaction. The reaction mixture was filtered, concentrated under vacuum and purified by flash chromatography to give the desired compounds (**5** and **6**).

#### N-isopropyl-3-(4-(N-phenylpropionamido)piperidin-1-yl)propanamide (**5**)

**Figure:**
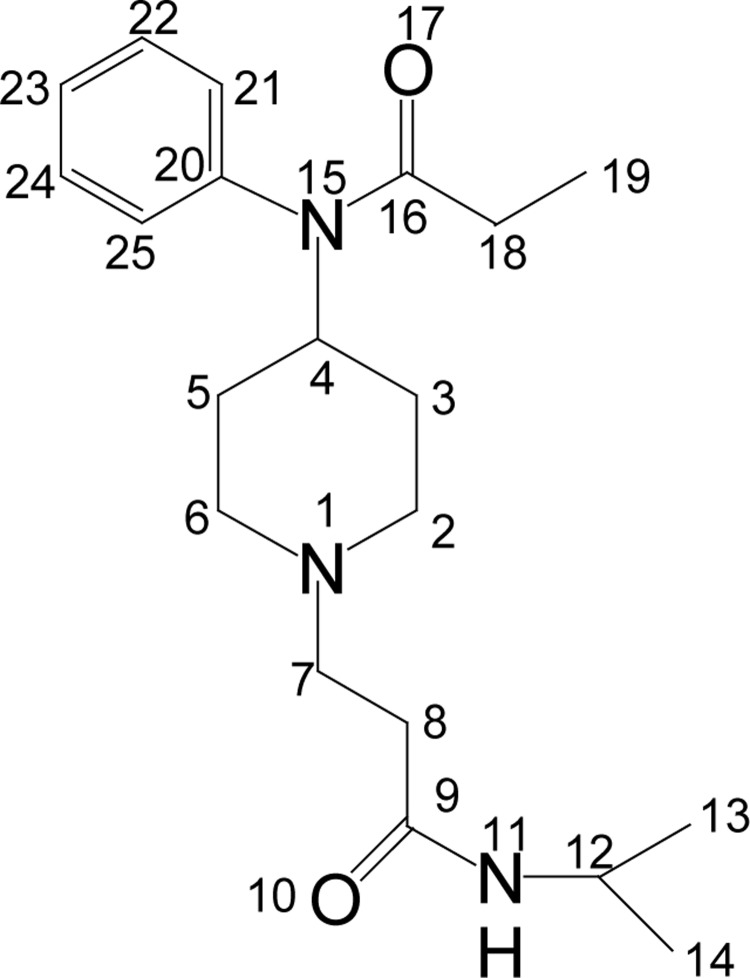


Colorless solid (60%); mp 108–110 °C. IR (KBr) _max_ 3327, 3235, 2941, 2822, 1657, 1626, 1460, 1448, 1264, 1283, 1123, 867, 643 cm^−1^; ^1^H NMR [CDCl_3_, 400 MHz): δ=0.90 (t, 3H, H-19), 1.10 (d, 6H, H-13, 14), 1.30 (dq, 2H, H-3_ax_, 5_ax_), 1.70 (bd, 2H, H-3_eq_, 5_eq_), 1.90 (q, 2H, H-18), 2.10 (bt, 2H, H-2_ax_, 6_ax_), 2.40 (t, 2H, H-8), 2.60 (t, 2H, H-7), 2.90 (bd, 2H, H-2_eq_, 6_eq_), 4.65 (m, 1H, H-4), 4.90 (m, 1H, H-12), 7.00 (d, 2 × Ar-H), 7.30 (m, 3×Ar-H); ^13^C NMR [CDCl_3_, 100MHz): d=9.6 (CH_3_, C-19), 22.7 (CH_3_, C-13, 14), 25.4 (CH_2_, C-18), 28.6 (CH_2_, C-3, 5), 30.6 (CH_2_, C-8), 32.4 (CH, C-12), 40.6 (CH_2_, C-2, 6), 52.0 (CH, C-4), 53.4 (CH_2_, C-7), 128.4 (CH, C-21, 25), 129.4 (CH, C-23), 130.3 (CH, C-22, 24), 138.8 (C, C-20), 171.4 (CO, C-16), 173.5 (CO, C-9); EI-MS m/z (pos): 345, 316, 288, 245, 231, 189, 175, 159, 146, 132, 120, 93, 82, 68, 55, 44, 29; Anal. Calcd. for C_20_H_31_N_3_O_2_: C, 69.45; H, 9.00; N, 12.12. Found: C, 69.53; H, 9.04; N, 12.16.

#### N-t-Butyl-3-(4-(N-phenylpropionamido)piperidin-1-yl)propanamide (**6**)

**Figure:**
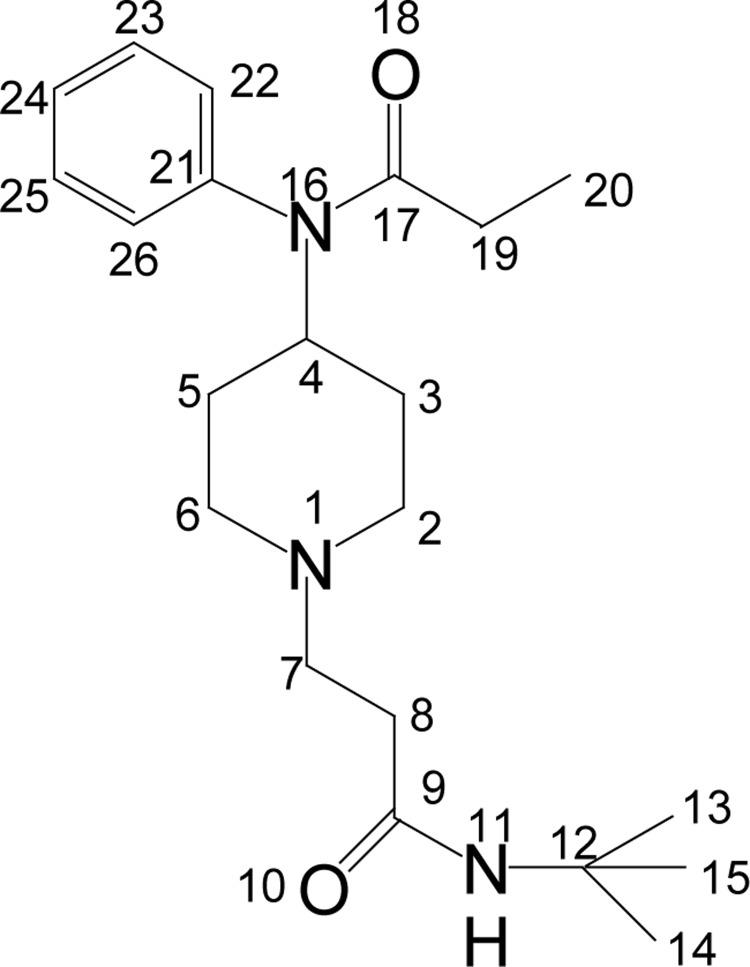


Colorless solid (65%); mp 92–95°C. IR (KBr) ν_max_ 3327, 3235, 3063, 2963, 2948, 2888, 1657, 1627, 1561, 1433, 1268, 1261, 1174, 837 cm^−1^; ^1^H NMR [CDCl_3_, 400 MHz): δ=1.00 (t, 3H, H-20), 1.13 (s, 9H, H-13, 14, 15), 1.40 (dq, 2H, H-3_ax_, 5_ax_), 1.70 (bd, 2H, H-3_eq_, 5_eq_), 1.90 (q, 2H, H-19), 2.10–2.30 (m, 4H, H-2_ax_, 6_ax_, 8), 2.50 (t, 2H, H-7), 2.90 (bd, 2H, H-2_eq_, 6_eq_), 4.65 (m, 1H, H-4), 7.10 (d, 2×Ar-H), 7.40 (m, 3×Ar-H), 8.20 (sbr, 1H, NH); ^13^C NMR [CDCl_3_, 100MHz): d=9.6 (CH_3_, C-20), 28.6 (CH_2_, C-19), 28.7 (CH_2_, C-3, 5), 30.6 (CH_3_, C-14, 14, 15), 33.4 (CH_2_, C-8), 50.2 (C, C-12), 51.8 (CH_2_, C-2, 6), 52.1 (CH, C-4), 53.7 (CH_2_, C-7), 128.4 (CH, C-22, 26), 129.3 (CH, C-24), 130.3 (CH, C-23, 25), 138.8 (C, C-21), 171.5 (CO, C-17), 173.5 (CO, C-9); EI-MS m/z (pos): 360, 340, 330, 302, 286, 259, 245, 231, 209, 189, 175, 159, 146, 132, 118, 96, 82, 57, 42, 29; Anal. Calcd. for C_21_H_33_N_3_O_2_: C, 70.12; H, 9.18; N, 11.58. Found: C, 70.16; H, 9.25; N, 11.69.

#### Synthesis of **7** and **8**

To a solution of Isopropanol/tert-butanol (38 mmol) in toluene (25 ml), 2-bromopropionyl chloride (38 mmol) was added drop-wise and the reaction mixture was stirred at room temperature for 4 h under nitrogen atmosphere. The reaction mixture was quenched with water and extracted with ethyl acetate. The organic phase was washed with saturated sodium bicarbonate solution and brine, dried over sodium sulfate and concentrated under reduced pressure to get the desired ester as oils. Thereafter, to a solution of isopropyl or t-butyl 2-bromopropionate (10 mmol) and *N*-(4-piperidinyl)propionanilide (10 mmol) in DMF-water, potassium carbonate was added and the reaction mixture was stirred at 80°C. When the reaction was complete, the reaction mixture was filtered, and the filtrate was diluted with water and extracted with ethyl acetate. The organic phase was separated, and the aqueous phase was extracted with ethyl acetate; the combined organic phase was washed with brine, dried over sodium sulfate and concentrated under vacuum to give the crude product which was purified by flash chromatography to give the desired compounds (**7** and **8**).

#### Isopropyl 2-[4-(N-phenylpropionamido)piperidin-1-yl]propionate (**7**)

**Figure:**
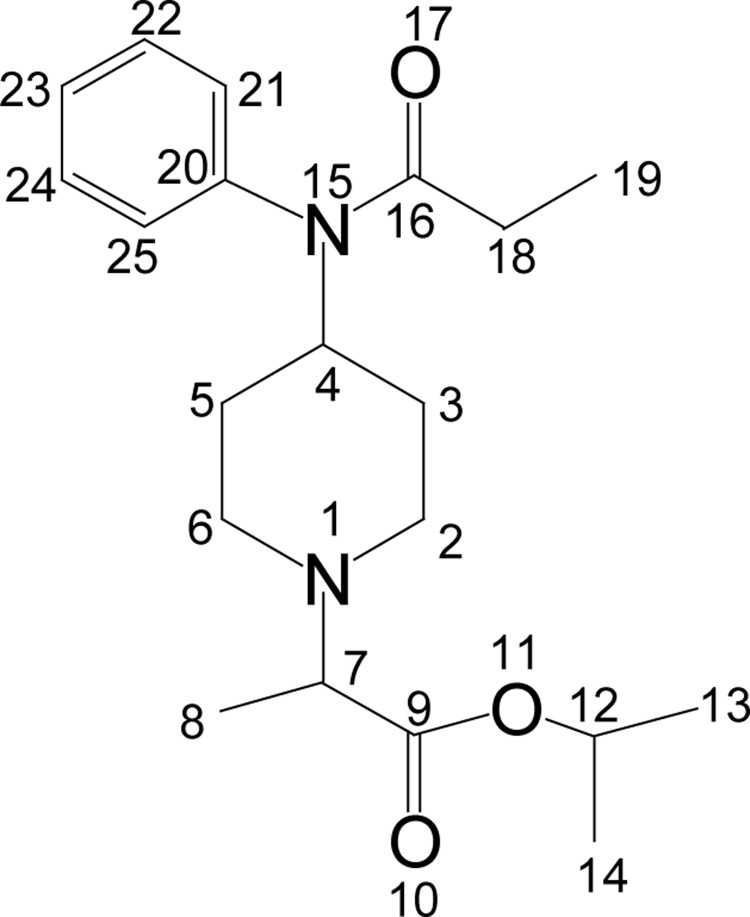


Colorless solid (76%); mp 67–69°C. IR (KBr) ν_max_ 3350, 3035, 2944, 2886, 1738, 1656, 1428, 1147, 929 cm^−1^; ^1^H NMR [CDCl_3_, 400 MHz): d=1.00 (t, 3H, H-19), 1.2–1.3 (m, 9H, H-8, 13, 14), 1.4–1.5 (m, 2H, H-3_ax_, 5_ax_), 1.75 (t, 2H, H-3_eq_, 5_eq_), 1.91 (q, 2H, H-18), 2.3–2.8 (m, 4H, H-2, 6), 3.15 (q, 1H, H-7), 4.65 (m, 1H, H-4), 5.0 (m, 1H, H-12), 7.1 (d, 2×Ar-H), 7.40 (m, 3×Ar-H); ^13^C NMR [CDCl_3_, 100MHz): δ=9.6 (CH_3_, C-19), 14.8 (CH_3_, C-8) 21.8 (CH_3_,C-13, 14), 22.0 (CH_2_, C-18), 28.5 (CH_2_, C-3, 5), 47.4 (CH_2_, C-2, 6), 50.9 (CH, C-4), 62.4 (CH, C-7), 68.4 (CH, C-12), 128.4 (CH, C-21, 25), 129.4 (CH, C-23), 130.3 (CH, C-22, 24), 138.7 (C, C-20), 171.6 (CO, C-16), 173.6 (CO, C-9); EI-MS m/z (pos): 346, 331, 317, 289, 259, 216, 203, 187, 146, 132, 110, 93, 82, 56; Anal. Calcd. for C_20_H_30_N_2_O_3_: C, 69.35; H, 8.67; N, 8.05. Found: C, 69.33; H, 8.73; N, 8.09.

#### t-butyl 2-[4-(N-phenylpropionamido)piperidin-1-yl]propionate (**8**)

**Figure:**
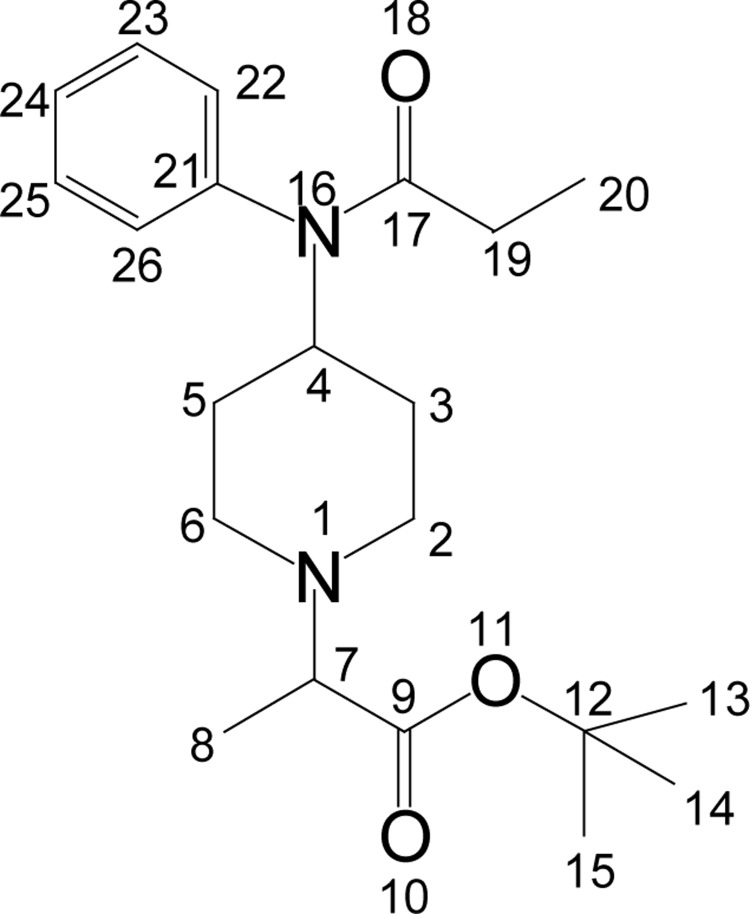


Colorless solid (74%); mp 98–100°C. IR (KBr) ν_max_ 3330, 3030, 2984, 2880, 1740, 1652, 1470, 1113, 1006, 929, 801; ^1^H NMR [CDCl_3_, 400 MHz): δ=1.00 (t, 3H, H-20), 1.18 (d, 3H, H-8), 1.34 (m, 2H, H-3_ax_, 5_ax_), 1.45 (s, 9H, H-13, 14, 15), 1.75 (m, 2H, H-3_eq_, 5_eq_), 1.90 (q, 2H, H-19), 2.35–2.47 (m, 2H, H- H-2_ax_, 6_ax_), 2.90 (bd, 2H, H-2_eq_, 6_eq_), 3.10 (q, 1H, H-7), 4.66 (m, 1H, H-4), 7.07 (d, 2×Ar-H), 7.38 (m, 3×Ar-H); ^13^C NMR [CDCl_3_, 100 MHz): δ=9.6 (CH_3_, C-20), 15.1 (CH_3_, C-8), 28.3 (CH_2_, C-19), 28.5 (CH_3_, C-13, 14, 15), 31.0 (CH_2_, C-3, 5), 47.4 (CH_2_, C-2, 6), 50.9 (CH, C-4), 52.4 (CH, C-7), 63.1 (C, C-12), 128.2 (CH, C-22, 26), 129.3 (CH, C-24), 130.5 (CH, C-23, 25), 138.9 (C, C-21), 172.4 (CO, C-17), 173.5 (CO, C-9); EI-MS m/z (pos): 360, 303, 289, 277, 259, 216, 203, 187, 156, 146, 132, 110, 93, 82, 56; Anal. Calcd. for C_21_H_32_N_2_O_3_: C, 70.02; H, 8.05; N, 7.58. Found: C, 69.97; H, 8.95; N, 7.77.

### Biological assay

#### Animals

Male Swiss albino mice (25–30 g) were procured from the Animal Facility of the Defence Research and Development Establishment (DRDE), Gwalior. The animals were housed in polypropylene cages on dust free rice husk as bedding material, with free access to food (Ashirwad Brand, Chandigarh, India) and water ad libitum. Prior to experiment, the animals were randomized and acclimatized for seven days in controlled environmental conditions (22±2°C; relative humidity 40–60%) at a 12h light/12 h dark cycle. The care and maintenance of the animals were as per the approved guidelines of the Committee for the Purpose of Control and Supervision of Experiments on Animals (CPCSEA), Ministry of Environment and Forest, Govt. of India, New Delhi, India. The experimental protocol was approved by the Institutional Ethical Committee on Animal Experimentations approved by CPCSEA.

#### Determination of LD_50_

The LD_50_ of the compounds was determined by intravenous (i.v.), intraperitoneal (i.p.) and oral (p.o.) routes following Dixon's up and down method (Dixon, 1965), using 4–6 mice for each value. All the compounds were dissolved in DMSO and administered in a volume <10ml/kg body weight. To determine the i.v. LD_50_, the compounds were administered through the tail vein using a 27 gauge needle, and for the p.o. LD_50_, a 16 gauge oral feeding cannula (HSE-Harvard, Germany) was used. Although, the animals were observed for 14 days, the mortality invariably occurred within the first 24h. All the dead animals were autopsied to see any visceral changes. All the observations were compared with those of fentanyl (Gupta *et al*., 2013).

#### Observational assessment

Observational assessment on spontaneous activities of the CNS, peripheral nervous system (PNS) and autonomic nervous system (ANS) was performed as per the modified method discussed elsewhere (Irwin, 1964, 1968). Mice were divided into five groups of twenty seven animals each as follows: (i) vehicle control (DMSO), (ii) **5**, (iii) **6**, (iv) **7** and (v) **8**. Three animals from each group received 0.25, 0.50, and 0.75 LD_50_ each of the compounds by i.v., i.p., and p.o. routes. Immediately after treatment, the animals were closely observed for 2 h by a blind observer for various CNS, PNS and ANS activities (Gupta *et al*., 2013).

#### Determination of opioid antagonist activity

To confirm the opioid receptor-mediated effects of the compounds, mice were treated with 0.50 LD_50_ (i.p.) of the analogs (**5–8**), in the presence or absence of naloxone hydrochloride (5.0 or 10.0 mg/kg; s.c.; –10 min). Soon after, the animals were closely observed for various neurotoxic manifestations of the compounds and their disappearance in the presence of naloxone hydrochloride (Matosiuk *et al.*, 2002; Leavitt, 2009). Three animals were used for each treatment.

#### Determination of ED_50_, potency ratio and therapeutic index

Analgesic ED_50_ of all the compounds was determined by formalin-induced hind paw licking method (Hunskar & Hole, 1987). The compounds were dissolved in DMSO and administered (i.p.) 30 min prior to administration of formalin (2.5%, 20 µl) injected sub-plantarly in one hind paw. The duration of paw licking as index of nociception was monitored at 0–5 min (first phase or neurogenic phase) and 15–30 min (second phase or inflammatory phase). Each compound was evaluated at four different doses, using four animals for each dose. Thereafter, Litchfield and Wilcoxon (1949) method was utilized for statistical evaluation of data and calculation of ED_50_ values. The potency ratio was determined as the ratio of ED_50_ of fentanyl and the ED_50_ of each analogue. The therapeutic index was calculated as the ratio of LD_50_ of the analogs and their corresponding ED_50_. All the values were compared with those of fentanyl (Gupta *et al.*, 2013).

#### Measurement of analgesic activity

Analgesic activity of the analogs was assessed at their ED_50_ by formalin-induced hind paw licking test (Hunskar & Hole, 1987) and tail immersion test (Janssen *et al.*, 1963). To perform the hind paw licking test, mice were divided into six groups of six animals each and given various treatments (i.p.) as follows: (i) vehicle control (DMSO), (ii) fentanyl (28.0 µg/kg, (iii) **5** (20.5 µg/kg), (iv) **6** (21.0 µg/kg), (v) **7** (35.5 µg/kg), and (vi) **8** (55.0 µg/kg). Thirty minutes after administration of different compounds, formalin (2.5%, 20 µl) was injected sub-plantarly in one hind paw. The duration of paw licking as index of nociception was monitored at 0–5min (neurogenic phase) and 15–30 min (inflammatory phase). To conduct the tail immersion test, mice received various treatments as discussed above. The distal part (2–3cm) of the tail was immersed in hot water maintained at 55.0±1.0°C, and the time taken by the mice to deflect or withdraw the tail was recorded as the reaction time. A cut off time of tail immersion was taken as 10 sec, and thereafter the measurement was stopped to avoid any tissue injury. The initial reading was taken immediately before treatment and then at 15, 30, 45, and 60 min post treatment. Tail withdrawal in vehicle treated mice usually occurs between 2.6 and 3.0 sec. Therefore, a withdrawal time of >3 sec was considered as a positive response. Prior to the analgesic test, the animals were screened by immersing their tail in hot water (55.0±1.0°C) and only those animals were selected for the experiment which showed tail withdrawal latency of <5 sec.

### Statistics

Results of analgesic activity were expressed as mean ± SE (n=6). The statistical analysis was carried out by Student's t test, using SigmaStat software (SSP Inc., USA). Statistical significance was drawn at p<0.05 and p<0.01.

## Results

### Toxicity of fentanyl analogs

Table 2 shows the LD_50_ values of fentanyl analogs by i.v., i.p. and p.o. routes in mice. All the analogs were found to be less toxic compared to fentanyl by all the routes of administration. The LD_50_ of fentanyl was 6.9, 17.5 and 27.8 mg/kg by i.v., i.p., and p.o. routes, respectively (Gupta *et al.*, 2013). On the basis of LD_50_, the order of toxicity of the compounds was: fentanyl >**7** >**8** >**6** >**5** for i.v. route, and fentanyl **>6 >5 >7 >8** for i.p. and p.o. routes. On the basis of LD_50_ by i.v. route, all the analogs showed more or less similar toxicity, but by i.p. and p.o. route, **7** and **8** were distinctly less toxic compared to **5** and **6**. Mice succumbing to lethal doses of the compounds were autopsied immediately, which revealed profuse intestinal hemorrhage.

**Table 2:** LD_50_ of fentanyl analogs by different routes in mice.

LD_50_ (mg/kg)			
Comp.	Intravenous	Intraperitoneal	Oral
**5**	57.0 (42.1–77.2)	113.8 (84.1–153.9)	285.8 (211.2–386.7)
**6**	45.3 (33.5–61.3)	107.3 (81.5–141.3)	220.7 (163.2–298.7)
**7**	35.0 (25.9–47.3)	277.9 (205.4–376.0)	717.9 (530.6–971.4)
**8**	44.0 (32.6–59.6)	349.9 (258.6–473.4)	903.9 (668.0–1223.0)

Fentanyl analogs, viz., 5-8 were administered through different routes and acute (24 h) LD_50_ was determined by Dixon's up and down method (Dixon, 1965). Values in parentheses are fiducial limits at 95% confidence interval.

### Observational assessment

Observational assessment based on CNS, PNS and ANS activities was made following administration of three doses of the compounds by i.v., i.p. and p.o. routes. The CNS activities included spontaneous motor activity, restlessness, grooming behavior, squatting, staggering, ataxic gait, lying flat on the belly, lying flat on the side, lying flat on the back, sleeping, narcosis, bizarre behavior, timidity, Straub′s phenomenon, writhing, tremors, twitches, opisthotonus, clonic convulsions, tonic convulsions, rolling and jumping and convulsions. The PNS activities (after manipulations) included auditory stimulus response, escape after touch, righting reflex, paresis of hind limbs, paresis of forepaws, and catalepsy in induced positions, while PNS activities (reflexes) included pinna reflex, corneal reflex, and pain following stimulation. The ANS activities included eyelids (closure or exophthalmus), salivation, lacrimation, cyanosis, piloerection, defecation and urination. All the compounds administered through i.v. (Table 3) and i.p. (Table 4) routes exhibited more intense activities compared to p.o. (Table 5) route. Also, all the compounds showed severe effects on CNS and PNS activities compared to ANS by all the routes of administration. Manifestations like defecation and micturation were only minimal. After p.o. administration, **5, 6** and **7** showed little CNS activity at low dose, but **8** did not show any response whatsoever. Also, all the analogs except **7** did not show any PNS activity (reflexes) at low and medium doses. After i.p. administration, none of the analogs revealed any PNS activity (reflexes) at low dose, while after i.v. administration all the analogs exhibited CNS and PNS activities at low dose, with the exception of **7**, which did not result in any PNS activity. In general, all the analogs exerted effects on CNS, PNS and ANS activities practically comparable to those induced by fentanyl.

**Table 3:** Observational assessment after intravenous administration of fentanyl analogs in mice.

			PNS		
Comp.	Dose	CNS	After manipulations	Reflexes	ANS
Control	–	0	0	0	0
5	Low	++	+	+	0
	Medium	+++	+++	++	+
	High	++++	++++	+++	+
6	Low	++	+	+	+
	Medium	+++	+++	+	+
	High	+++	++++	++	+
7	Low	+	0	0	+
	Medium	++	+	+	+
	High	+++	++	++	+
8	Low	++	++	+	+
	Medium	+++	+++	++	+
	High	++++	+++	+++	++

Mice were intravenously administered 0.25 (Low), 0.50 (Medium), and 0.75 (High) LD_50_ of fentanyl analogs, viz., 5-8. The control animals received DMSO. Immediately after treatment, the animals were closely observed for 2 h by a blind observer for its effects on CNS, PNS (effects after manipulation and effects on reflexes) and ANS activities. The scorings were given as: 0 (no observational change), + (little activity), ++ (moderate flexibility), +++ (strong response) and ++++ (exaggerated response). Each treatment included three animals.

**Table 4:** Observational assessment after intraperitoneal administration of fentanyl analogs in mice.

			PNS		
Comp.	Dose	CNS	After manipulations	Reflexes	ANS
Control	–	0	0	0	0
5	Low	+	+	0	0
	Medium	++	+	+	+
	High	+++	+++	++	+
6	Low	+	+	0	0
	Medium	++	++	0	0
	High	+++	+++	++	+
7	Low	+	+	0	+
	Medium	++	++	+	+
	High	++	+++	+	++
8	Low	++	+	0	+
	Medium	++	+++	++	+
	High	+++	+++	++	+

Mice were intraperitoneally administered 0.25 (Low), 0.50 (Medium), and 0.75 (High) LD_50_ of fentanyl analogs, viz., 5-8. The control animals received DMSO. Immediately after treatment, the animals were closely observed for 2 h by a blind observer for its effects on CNS, PNS (effects after manipulation and effects on reflexes) and ANS activities. The scorings were given as: 0 (no observational change), + (little activity), ++ (moderate flexibility), +++ (strong response) and ++++ (exaggerated response). Each treatment included three animals.

**Table 5:** Observational assessment after oral administration of fentanyl analogs in mice.

			PNS		
Comp.	Dose	CNS	After manipulations	Reflexes	ANS
Control	–	0	0	0	0
5	Low	+	+	0	0
	Medium	++	+	0	+
	High	+++	+++	+	+
6	Low	+	+	0	+
	Medium	+	++	0	+
	High	+++	+++	++	+
7	Low	+	+	0	0
	Medium	++	++	+	+
	High	++	+++	++	+
8	Low	0	+	0	+
	Medium	+	+	0	+
	High	+++	+++	++	+

Mice were orally administered 0.25 (Low), 0.50 (Medium), and 0.75 (High) LD_50_ of fentanyl analogs, viz., 5–8. The control animals received DMSO. Immediately after treatment, the animals were closely observed for 2 h by a blind observer for its effects on CNS, PNS (effects after manipulation and effects on reflexes) and ANS activities. The scorings were given as: 0 (no observational change), + (little activity), ++ (moderate flexibility), +++ (strong response) and ++++ (exaggerated response). Each treatment included three animals.

### Determination of opioid antagonist activity

In the present study, pre-treatment of 5 mg/kg naloxone was found to quickly reverse the neurotoxic effects produced by 0.50 LD_50_ (i.p.) of **5** and **6**, while the same dose of naloxone could not reverse the neurotoxic effects of **7** and **8**, for which a higher dose of 10 mg/kg was required.

### Determination of ED_50_, potency ratio and therapeutic index

Table 6 summarizes the results on analgesic ED_50_, potency ratio and therapeutic index of fentanyl analogs, determined by formalin-induced hind paw licking method. The ED_50_ (µg/kg) of **5** (20.5) and **6** (21.0) was less, and that of **7** (35.5) and **8** (55.0) was more than that of fentanyl (28.0). Conversely, the potency ratio of **5** (1.37) and **6** (1.33) was more, and that of **7** (0.79) and **8** (0.51) was less than that of fentanyl (1.0). The therapeutic index was in the following order: **7** (7828.2) >**8** (6361.8) >**5** (5551.2) >**6** (5109.5) >fentanyl (625.0). In brief, the lowest ED_50_ and the highest potency ratio were observed in the case of **5,** followed by **6**. However, the maximum therapeutic index was exhibited by **7,** compared to the lowest observed in case of fentanyl. This indicates that all the compounds were 8–12 times safer than fentanyl.

**Table 6:** ED_50_, potency ratio and therapeutic index of fentanyl analogs in mice.

Comp.	ED_50_ (µg/kg)	Potency ratio	Therapeutic index
**5**	20.5 (11.3–37.1)	1.37 (1.32–1.41)	5551.2 (4148.2–7442.5)
**6**	21.0 (11.4–38.6)	1.33 (1.31–1.36)	5109.5 (3660.6–7149.1)
**7**	35.5 (17.4–72.4)	0.79 (0.86–0.72)	7828.2 (5193.4–11804.6)
**8**	55.0 (32.9–91.9)	0.51 (0.45–0.57)	6361.8 (5151.3–7860.2)

Analgesic ED_50_ of fentanyl analogs, viz., 5–8 was determined by formalin-induced hind paw licking method (Hunskar & Hole, 1987; Litchfield & Wilcoxon, 1949). The potency ratio was determined as the ratio of ED_50_ of fentanyl 28.0 (µg/kg) (Gupta *et al.*, 2013) and ED_50_ of each analog. The therapeutic index was calculated as the ratio of LD_50_ of the compounds and their ED_50_. Values in parentheses are fiducial limits at 95% confidence interval.

### Measurement of analgesic activity

**Figure 1:**
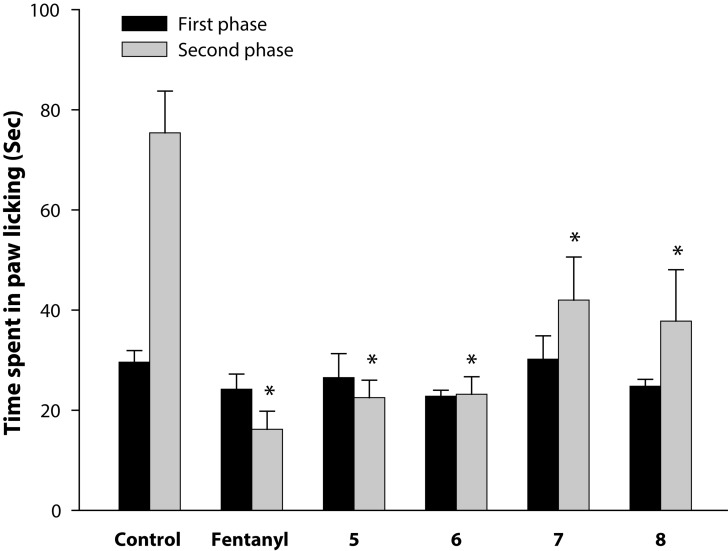
Analgesic activity of fentanyl and its four analogs, viz. **5–8,** was measured by formalin-induced hind paw licking method in mice. Values are expressed as mean ± SE (n=6). *p<0.01 (Student's *t* test).

**Figure 2:**
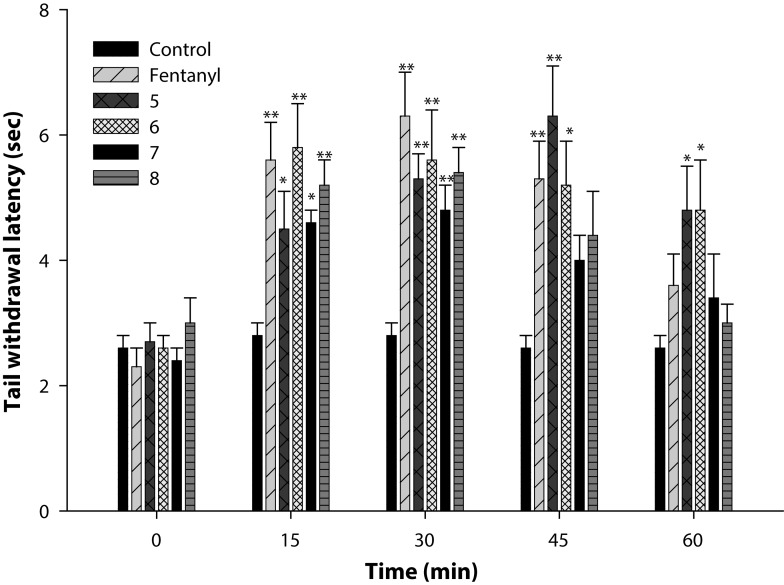
Analgesic activity of fentanyl and its four analogs, viz. **5-8,** was measured by tail immersion test. Values are expressed as mean ± SE (n=6). *p< 0.05 and **p< 0.01 (Student's *t* test).

Figure 1 shows the analgesic activity of fentanyl and its analogs measured at their respective ED_50_, determined by formalin-induced hind paw licking method. The duration of paw licking as index of nociception was observed at the first phase (0–5 min) and second phase (15–30 min). In the first phase, none of the compounds displayed any analgesic activity because the time spent in paw licking did not decrease compared to control. However, in the second phase, all the compounds exhibited significant (p<0.01) analgesic activity compared to control. Further, compared to control, the percentage of decrease in time of paw licking was 78.5, 68.8, 69.2, 44.3, and 49.9 for fentanyl, **5**, **6**, **7**, and **8**, respectively. The data show that in the second phase, antinociceptive activity of **7** and **8** was lower than that of other compounds. Figure 2 refers to the analgesic activity of fentanyl and its analogs measured by tail immersion method. Animals receiving ED_50_ of the compounds were observed to increase the reaction time after thermal stimuli given at different time points after treatment. All the values were compared to control at the corresponding time point. Fentanyl showed significant (*p*<0.01) analgesic activity at 15, 30 and 45 min post-treatment, the maximum being at 30 min. The analgesic activity of **5** and **6** was evident at all the time points. The analgesic activity of **5** progressively increased after 15 min to a maximum at 45 min, thereafter it declined at 60 min. On the other hand, analgesic activity of **6** was maximum at 15 min, which gradually declined by 60 min. However, at this time also both **5** and **6** were significantly (*p*<0.05) different from the corresponding control. Compound **7** and 8 displayed significant analgesic activity at 15 and 30 min only, the maximum for both being observed at 30 min. In brief, both **5** and **6** demonstrated longer antinociceptive activity compared to fentanyl, while the effects of **7** and **8** were short-lived.

## Discussion

Although fentanyl is widely used as a narcotic analgesic agent, it has been implicated in drug abuse and fatalities due to overdosing and narrow therapeutic window (Yassen *et al.*, 2008; Jumbelic, 2010). Over many recent years, SAR and molecular modeling of several new analogs of fentanyl have projected many potent compounds (Vučković *et al.*, 2000). Our recent work also revealed some effective 1-substituted analogues of fentanyl (Gupta *et al.*, 2013). The present study reports the synthesis, and biological evaluation of four new fentanyl analogs. The LD_50_ of the new analogs by different routes revealed that all the compounds were less toxic compared to fentanyl, thus showing an improved margin of safety over fentanyl. Autopsy of the animals succumbing to high doses of the compounds showed severe intestinal hemorrhage. This possibly occurred due to pooling of blood following hypovolemic shock. Similar observations were also made during our previous study (Gupta *et al.*, 2013) and after administration of methyl-substituted and para-substituted fentanyl analogs (Higashikawa & Suzuki, 2008).

Observational assessment on spontaneous CNS, PNS, and ANS activities is usually performed to evaluate the psychotropic activity and toxicity of the compounds (Irwin, 1964, 1968). Observational assessment made in the present study revealed that all the compounds exerted significant dose-dependent influence on CNS and PNS activities. Also, the compounds were found to induce straub's phenomenon, catalepsy, rigidity, circling and stereotypical behavior, which are distinctive characteristics of opioid analgesics. Severe convulsions, a typical attribute of morphine intoxication, were also observed. This can be attributed to inhibition of release of gamma-aminobutyric acid by interneurons (McGinty & Friedman, 1988). All the compounds were evaluated through parenteral and p.o. routes of administration, which are the preferred routes of opioids for pain management (Gardocki & Yelnosky, 1964; Hallenbeck, 2003; Vuckovic *et al.*, 2011). In the present study, most of the observations on motor coordination and behavioral tests of fentanyl analogs were very similar to previous observations with fentanyl (Gardocki & Yelnosky, 1964). There were also reduced ANS activities, like defecation and micturation, which are typical of opioid analgesics (Gupta *et al.*, 2013).

Short acting opioid antagonists, such as naloxone, have been successfully used to rapidly reverse the neurotoxic effects of opioid overdose (Leavitt, 2009). Naloxone is a nonselective antagonist of opioid receptors, and is generally used to verify any opioid-mediated effects of the drugs (Jagerovic *et al.*, 2002). In the present study, pre-treatment of naloxone completely reversed the neurotoxic effects of all the analogs, confirming that their effects were possibly mediated through MOR (Mićović et al., 2000; Jagerovic *et al.*, 2002; Leavitt, 2009). This is in agreement with a previous study which reported that such a receptor is involved in Straub's phenomenon, muscle rigidity, catalepsy and other morphine-like behavioral effects in rats (Vučković et al., 2012).

In the present study, the analgesic activity of 1-substituted analogs of fentanyl was determined by formalin-induced hind paw licking method (Hunskar & Hole, 1987) and tail immersion test (Janssen *et al.*, 1963). The formalin test is a widely used model for screening novel compounds for the treatment of neuropathic pain. The method involves a behavioral nociceptive test that assesses the response of the animal to moderate and continuous pain (Meunier *et al.*, 1998). Formalin produces biphasic pain behavior. The first phase (i.e. neurogenic phase) is due to the direct effect of formalin on nociceptors, while the second phase (i.e. inflammatory phase) is due to the development of an inflammatory response caused by tissue injury leading to the release of histamine, serotonin, prostaglandin and excitatory amino acids (Correa & Calixto, 1993; Damas & Liegeois, 1999). In the present study, all the analogs were found to be more effective in the second phase, which could be due to their implications as inhibitors of pain mediators during the late phase. In the present study, **5** and **6** exhibited higher potency compared to fentanyl but lower analgesic activity when evaluated at respective ED_50_. Potency and efficacy are different concepts, and when an agonist possesses high potency, it need not display also high efficacy, and vice versa (Lambert, 2004). An agonist capable of producing the maximum response in that system is termed a full agonist and anything that produces a lower response is a partial agonist. The ability of the agonist to bind to the receptor will determine the ability to produce a response and to some extent the size of that response (Lambert, 2004). The tail immersion test is widely employed for opioid analgesics. This method gives intensity, onset, peak, duration of action and safety of fentanyl and other morphine like analgesics (Janssen *et al.*, 1963). In the present study, onset, peak and duration of the analgesic effect of all the analogs were compared with those of fentanyl by using tail immersion test. In order to perform this study, all the compounds were tested at their ED_50_. We found that **5** and **6** produced analgesia for a longer duration compared to fentanyl. Most of the opioid analgesics exert their analgesic and adverse effects primarily through MOR. However, individual strong opioids may interact, at least in part, with different opioid receptor sub-populations or modulate MOR signaling in different ways (Pasternak, 2004; Lee *et al.*, 2007), which may improve tolerability (Ananthan, 2006; Smith, 2008; Spetea *et al.*, 2010). Previous studies have shown that 4-methyl fentanyl was four times more potent than fentanyl (Mićović et al., 2000), while introduction of 3-carbomethoxy group in the piperidine ring of fentanyl reduced the potency but did not affect the tolerability and safety (Vučkovic *et al.*, 2011). The shorter duration of action of 3-carbomethoxy fentanyl in comparison with fentanyl might be due to the susceptibility of the carbomethoxy group to rapid hydrolysis by non-specific esterases (Feldman *et al.*, 1991). It is also conceivable that the introduction of a 3-carbomethoxy group in the piperidine ring affects the duration of action by altering physicochemical properties (Scholz *et al*., 1996).

## Conclusion

Opioid analgesics are usually prescribed for acute and chronic pain management but their use is restricted due to undesirable side effects and a narrow therapeutic window. The present study addresses the synthesis and biological evaluation of four new 1-substituted analogs of fentanyl, where the phenethyl tail of fentanyl was replaced by different functional groups. All the compounds exhibited 8–12 fold increase in therapeutic index but only two compounds (**5** and **6**) produced lower ED_50_ and higher potency ratio compared to fentanyl. Thus out of the four compounds tested, only two were found to be promising for further studies on pain management.

## References

[CIT0001] Ananthan S (2006). Opioid ligands with mixed mu/delta opioid receptor interactions: an emerging approach to novel analgesics. AAPS J.

[CIT0002] Bagley JR, Kudzma LV, Lalinde NL, Colapret JA, Huang B-S, Lin B-S, Jerussi TP, Benvenga MJ, Doorley BM, Ossipov MH, Spaulding TC, Spencer HK, Rudo FG, Wynn RL (1991). Evolution of the 4-anilidopiperidine class of opioid analgesics. Med Res Rev.

[CIT0003] Bi-Yi C, Wen-Qiao J, Jie C, Xin-Jian C, You-Cheng Z, Zhi-Qiang C (1999). Analgesic activity and selectivity of isothiocyanate derivatives of fentanyl analogs for opioid receptors. Life Sci.

[CIT0004] Casy AF, Parfitt RT (1986). Opioid analgesics.

[CIT0005] Correa CR, Calixto JB (1993). Evidence for participation of B1 and B2 kinin receptors in formalin-induced nociceptive response in the mouse. Br J Pharmacol.

[CIT0006] Damas J, Liegeois JF (1999). The inflammatory reaction induced by formalin in the rat paw. Naunyn-Schmeidebergs Arch Pharmacol.

[CIT0007] Dixon WJ (1965). The up-and-down method for small samples. J Amer Statist Assoc.

[CIT0008] Feldman PL, James MK, Brackeen MF, Bilotta JM, Schuster SV, Lahey AP, Lutz MW, Johnson MR, Leighton HJ (1991). Design, synthesis, and pharmacological evaluation of ultrashort- to long-acting opioid analgetics. J Med Chem.

[CIT0009] Gardocki JF, Yelnosky J (1964). A study of some of the pharmacological actions of fentanyl citrate. Toxicol Appl Pharmacol.

[CIT0010] Gerak LR, Moerschbaecher JM, Bagley JR, Brockunier LL, France CP (1999). Effects of a novel fentanyl derivative on drug discrimination and learning in rhesus monkeys. Pharmacol Biochem Behav.

[CIT0011] Gupta PK, Yadav SK, Bhutia YD, Singh P, Rao P, Gujar NL, Ganesan K, Bhattacharya R (2013). Synthesis and comparative bioefficacy of N-(1-phenethyl-4-piperidinyl)propionanilide (fentanyl) and its 1-substituted analogs in Swiss albino mice. Med Chem Res.

[CIT0012] Hallenbeck JL (2003). Palliative care perspective.

[CIT0013] Higashikawa Y, Suzuki S (2008). Studies on 1-(2-phenethyl)-4-(N-propionylanilino)piperidine (fentanyl) and its related compounds. VI. Structure-analgesic activity relationship for fentanyl, methyl-substituted fentanyls and other analogues. Forensic Toxicol.

[CIT0014] Hunskar S, Hole K (1987). The formalin test in mice: dissociation between inflammatory and non-inflammatory pain. Pain.

[CIT0015] Irwin S, Nodin JH, Siegler PE (1964). Drug screening and evaluation of new compounds in animals. Animal and clinical techniques in drug evaluation.

[CIT0016] Irwin S (1968). Comprehensive observational assessment: Ia. A systematic, quantitative procedure for assessing the behavioural and physiologic state of the mouse. Psychopharmacologia (Berl).

[CIT0017] Jagerovic N, Cano C, Elguero J, Goya P, Callado LF, Meana JJ, Girón R, Abalo R, Ruiz D, Goicoechea C, Martín MA (2002). Long-Acting Fentanyl Analogues: Synthesis and Pharmacology of N-(1-Phenylpyrazolyl)-N-(1-phenylalkyl-4-piperidyl)propanamides. Bioorg Med Chem.

[CIT0018] Janssen PAJ, Niemegeers CJE, Dony JGH (1963). The inhibitory effect of fentanyl and other morphine-like analgesics on the warm water induced tail withdrawal reflex in rats. Arzneimittel-Forsch-Drug Res.

[CIT0019] Jumbelic MI (2010). Deaths with transdermal fentanyl patches. Am J Forensic Med Pathol.

[CIT0020] Kalso E, Edwards JE, Moore RA, McQuay HJ (2004). Opioids in chronic non-cancer pain: systematic review of efficacy and safety. Pain.

[CIT0021] Kieffer BL, Evans CJ (2009). Opioid receptors: from binding sites to visible molecules in Vivo. Neuropharmacology.

[CIT0022] Kieffer BL (1995). Recent advances in molecular recognition and signal transduction of active peptides: receptors for opioid peptides. Cell Mol Neurobiol.

[CIT0023] Lambert DG (2004). Drugs and receptors. Contin Educ Anaesth Crit Care Pain.

[CIT0024] Leavitt SB (2009). Opioid antagonists, naloxone & naltrexene-Aids for pain management. An overview of clinical evidence.

[CIT0025] Lee YS, Nyberg J, Moye S, Agnes RS, Davis P, Ma SW, Lai J, Porreca F, Vardanyan R, Hruby VJ (2007). Understanding the structural requirements of 4-anilidopiperidine analogues for biological activities at mu and delta opioid receptors. Bioorg Med Chem Lett.

[CIT0026] Lemmens H (1995). Pharmacokinetic-pharmacodynamic relationships for opioids in balanced anaesthesia. Clin Pharmacokinet.

[CIT0027] Litchfield JT, Wilcoxon F (1949). A simplified method of evaluating dose-effect experiments. J Pharm Exp Ther.

[CIT0028] Mather LE (1983). Clinical pharmacokinetics of fentanyl and its newer derivatives. Clin Pharmacokinet.

[CIT0029] Matosiuk D, Fidecka S, Antkiewicz-Michaluk L, Lipkowski J, Dybala I, Koziol AE (2002). Synthesis and pharmacological activity of new carbonyl derivatives of 1-aryl-2-iminoimidazolidine. Part 2. Synthesis and pharmacological activity of 1,6-diaryl-5,7(1H)dioxo-2,3-dihydroimidazo[1,2-a][1,3,5]triazines. Eur J Med Chem.

[CIT0030] Mayes S, Ferrone M (2006). Fentanyl HCl patient-controlled iontophoretic transdermal system for the management of acute postoperative pain: Summary/formulary recommendation. Ann Pharmacother.

[CIT0031] McGinty J, Friedman D (1988). Opioids in the hippocampus. Natl Inst Drug Abuse Res Monogr Ser.

[CIT0032] Meunier CJ, Burton J, Cumps J, Verbeeck RK (1998). Evaluation of the formalin test to assess the analgesic activity of diflunisal in the rat. Eur J Pharm Sci.

[CIT0033] Mićović IV, Ivanović MD, Vuckovic SM, Prostran MŠ, Došen-Mićović L, Kiricojević VD (2000). The synthesis and preliminary pharmacological evaluation of 4-methyl fentanyl. Bioorg Med Chem Lett.

[CIT0034] Pasternak GW (2004). Multiple opiate receptors: déjà vu all over again. Neuropharmacology.

[CIT0035] Portoghese PS (1992). The role of concepts in structure-activity-relationship studies of opioid ligands. J Med Chem.

[CIT0036] Scholz J, Steinfath M, Schulz M (1996). Clinical pharmacokinetics of alfentanyl, fentanyl and sufentanyl. An update. Clin Pharmacokinet.

[CIT0037] Smith MT (2008). Differences between and combinations of opioids re-visited. Curr Opin Anaesthesiol.

[CIT0038] Spetea M, Bohotin CR, Asim MF, Stübegger K, Schmidhammer H (2010). In vitro and in vivo pharmacological profile of the 5-benzyl analogue of 14-methoxymetopon, a novel mu opioid analgesic with reduced propensity to alter motor function. Eur J Pharm Sci.

[CIT0039] Vogel HG (2002). Psychotropic and neurotropic activity. Drug Discovery and Evaluation. Pharmacological Assays.

[CIT0040] Van Nimmen NFJ, Poels KLC, Veulemans HAF (2004). Highly sensitive gas chromatographic-mass spectrometric screening method for the determination of picogram levels of fentanyl, sufentanil and alfentanil and their major metabolites in urine of opioid exposed workers. J Chromatogr B.

[CIT0041] Vučkovic S, Prostran M, Ivanović M, Dosen-Mićović L, Vujović KS, Vucetic C, Kadija M, Mikovic Z (2011). Pharmacological evaluation of 3-carbomethoxy fentanyl in mice. Pharmaceuticals.

[CIT0042] Vučkovic S, Prostran M, Ivanović M, Ristović Z, Stojanović R (2000). Antinociceptive activity of the novel fentanyl analogue iso-carfentanil in rats. Jpn J Pharmacol.

[CIT0043] Vučkovic S, Savić VK, Ivanović M, Došen-Mićović L, Todorovic Z, Vučetić Č, Prostran M, Prostran Milica (2012). Neurotoxicity evaluation of fentanyl analogs in rats. Acta Veterinaria.

[CIT0044] Yassen A, Olofsen E, Kan J, Dahan A, Danhof M (2008). Pharmacokinetic-pharmacodynamic modeling of the effectiveness and safety of buprenorphine and fentanyl in rats. Pharmaceut Res.

